# Supplementary material for: Differences in the Tumor Molecular and Microenvironmental Landscape between Early (Non-Metastatic) and De Novo Metastatic Primary Luminal Breast Tumors
Source: Cancers (Basel). 2023 Aug 30;15(17):4341. doi: 10.3390/cancers15174341 (PMC10486668; doi:10.3390/cancers15174341)
Supplement: Supplementary file 1 [file cancers-15-04341-s001.zip › Supplementary Figure S1.pdf]

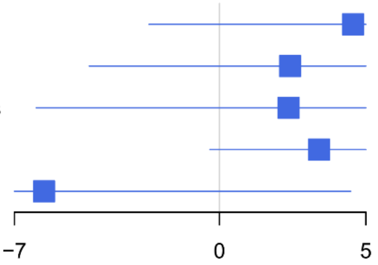

	coef (95% CI)	p-value	FDR
sTILs	4.56 (−2.40, 11.53)	0.189	0.415
Immune cells	2.41 (−4.45, 9.27)	0.474	0.576
Tumour epithelial cells	2.36 (−6.26, 10.98)	0.576	0.576
Normal epithelial cells	3.40 (−0.33, 7.13)	0.072	0.360
Fibroblasts	−5.99 (−16.46, 4.49)	0.249	0.415

Supplementary Figure S1: Forest plot of the continuous pathological cellular characteristics (sTILs, immune cells, tumor epithelial cells, normal epithelial cells, and fibroblasts) from the multivariable model corrected for cT and grade compared between dnMBC and eBC. *95% confidence interval was added to show the performance of this model. The coefficient gives the direction of the outcome on the variable. A positive coefficient value indicates a higher outcome in the dnMBC group and vice versa for a negative coefficient value. P-values were calculated with the Wald test and adjusted for multiple testing using the Benjamini-Hochberg method when required. CI: confidence interval; coef: coefficient; dnMBC: de novo metastasized breast tumor group; eBC: non-primary metastatic breast tumor group; FDR: false discovery rate; sTILs: stromal tumor infiltrating lymphocytes.*
