# Supplementary material for: Differences in the Tumor Molecular and Microenvironmental Landscape between Early (Non-Metastatic) and De Novo Metastatic Primary Luminal Breast Tumors
Source: Cancers (Basel). 2023 Aug 30;15(17):4341. doi: 10.3390/cancers15174341 (PMC10486668; doi:10.3390/cancers15174341)
Supplement: Supplementary file 1 [file cancers-15-04341-s001.zip › Supplementary Figure S2.pdf]

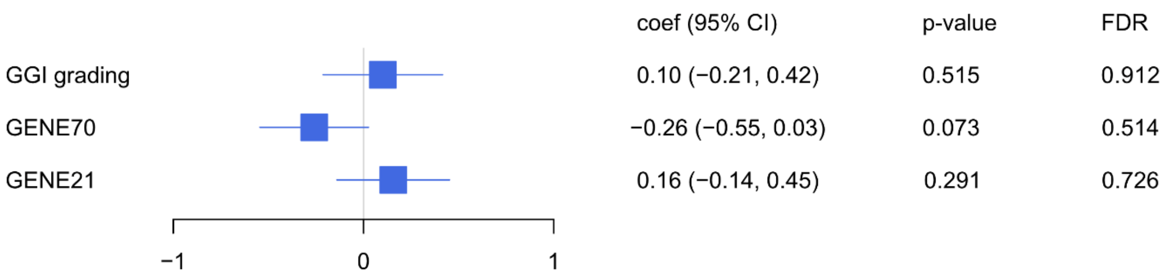

Supplementary Figure S2: Forest plot of the gene expression signatures (GGI grading, GENE21, and GENE70) from the multivariable model corrected for cT and grade compared between dnMBC and eBC. *95% confidence interval was added to show the performance of this model. The coefficient gives the direction of the outcome on the variable. A positive coefficient value indicates a higher outcome in the dnMBC group and vice versa for a negative coefficient value. P-values were calculated with the Wald test and adjusted for multiple testing using the Benjamini-Hochberg method when required. CI: confidence interval; coef: coefficient; dnMBC: de novo metastasized breast tumor group; eBC: non-primary metastatic breast tumor group; FDR: false discovery rate; GGI: genomic grade index.*
