# Supplementary material for: Differences in the Tumor Molecular and Microenvironmental Landscape between Early (Non-Metastatic) and De Novo Metastatic Primary Luminal Breast Tumors
Source: Cancers (Basel). 2023 Aug 30;15(17):4341. doi: 10.3390/cancers15174341 (PMC10486668; doi:10.3390/cancers15174341)
Supplement: Supplementary file 1 [file cancers-15-04341-s001.zip › Supplementary Figure S4.pdf]

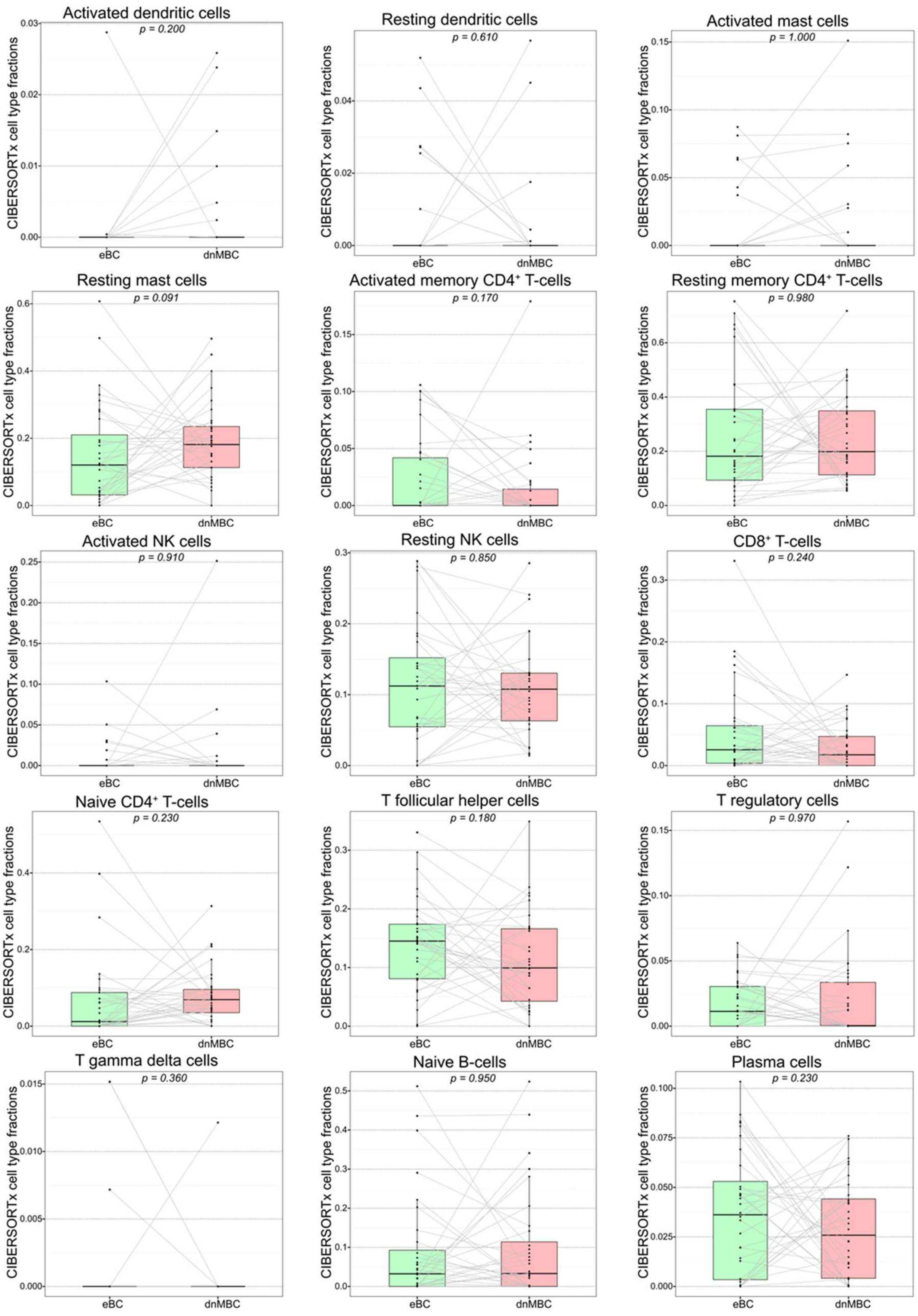

Supplementary Figure S4: Integrated boxplots of paired Wilcoxon analysis of cell type fractions in the CIBERSORTx software between eBC and dnMBC group*. P-values are FDR-corrected. CD: cluster of differentiation; dnMBC: de novo metastasized breast tumor group; eBC: non-primary metastatic breast tumor group; FDR: false discovery rate; NK: natural killer.*
