# Supplementary material for: Differences in the Tumor Molecular and Microenvironmental Landscape between Early (Non-Metastatic) and De Novo Metastatic Primary Luminal Breast Tumors
Source: Cancers (Basel). 2023 Aug 30;15(17):4341. doi: 10.3390/cancers15174341 (PMC10486668; doi:10.3390/cancers15174341)
Supplement: Supplementary file 1 [file cancers-15-04341-s001.zip › Supplementary Figure S6.pdf]

	coef (95% CI)	p-value	FDR
AKT_MTOR_MG.up	0.31 (0.03, 0.59)	0.032	0.457
IFNA.down	0.22 (−0.09, 0.53)	0.157	0.548
IFNG.down	0.47 (0.16, 0.79)	0.004	0.135
Memory B-cells	0.03 (0.00, 0.06)	0.094	0.514
Neutrophils	0.00 (0.00, 0.00)	0.329	0.725

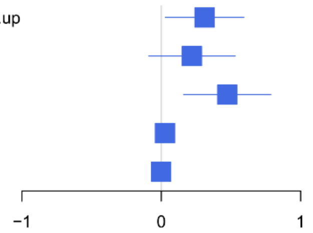

Supplementary Figure S6: Forest plot of the immunity-related CIBERSORTx immune cell types (memory B-cells and neutrophils) and signatures (AKT_MTOR_MG.up, IFNA.down and IFNG.down) from the multivariable model corrected for cT and grade compared between dnMBC and eBC. *95% confidence interval was added to show the performance of this model. The coefficient gives the direction of the outcome on the variable. A positive coefficient value indicates a higher outcome in the dnMBC group and vice versa for a negative coefficient value. P-values were calculated with the Wald test and adjusted for multiple testing using the Benjamini-Hochberg method when required. CI: confidence interval; coef: coefficient; dnMBC: de novo metastasized breast tumor group; eBC: non-primary metastatic breast tumor group; FDR: false discovery rate.*
