# Supplementary material for: Differences in the Tumor Molecular and Microenvironmental Landscape between Early (Non-Metastatic) and De Novo Metastatic Primary Luminal Breast Tumors
Source: Cancers (Basel). 2023 Aug 30;15(17):4341. doi: 10.3390/cancers15174341 (PMC10486668; doi:10.3390/cancers15174341)
Supplement: Supplementary file 1 [file cancers-15-04341-s001.zip › Supplementary Figure S7.pdf]

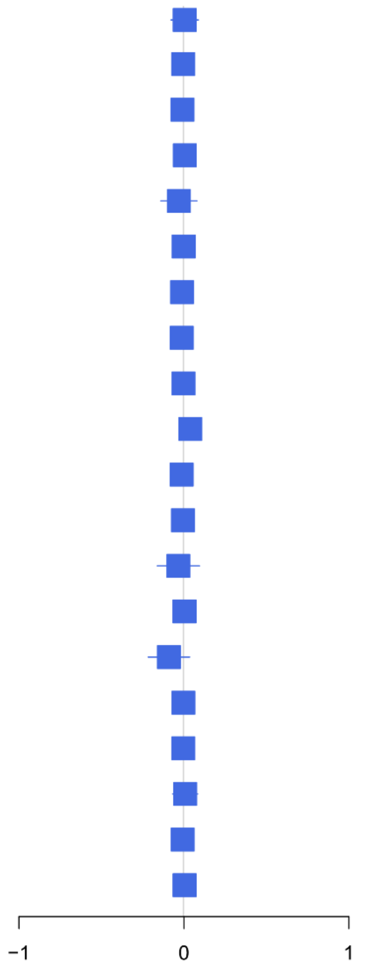

	coef (95% CI)	p-value	FDR
Naive B-cells	0.01 (−0.08, 0.09)	0.848	0.912
Plasma cells	0.00 (−0.02, 0.02)	0.912	0.912
CD8$^+$ T-cells	−0.01 (−0.04, 0.03)	0.743	0.912
CD4$^+$ Naive T-cells	0.01 (−0.05, 0.07)	0.767	0.912
Resting memory CD4$^+$ T-cells	−0.03 (−0.14, 0.08)	0.614	0.912
Activated memory CD4$^+$ T-cells	0.00 (−0.02, 0.02)	0.854	0.912
T follicular helper cells	−0.01 (−0.05, 0.04)	0.703	0.912
T regulatory cells	−0.01 (−0.03, 0.01)	0.218	0.653
T gamma delta cells	0.00 (0.00, 0.00)	0.363	0.726
Resting NK cells	0.04 (0.00, 0.08)	0.046	0.457
Activated NK cells	−0.01 (−0.03, 0.01)	0.305	0.726
Monocytes	0.00 (−0.03, 0.02)	0.812	0.912
M0 Macrophages	−0.03 (−0.16, 0.10)	0.628	0.912
M1 Macrophages	0.01 (−0.01, 0.02)	0.345	0.726
M2 Macrophages	−0.09 (−0.21, 0.04)	0.164	0.548
Resting dendritic cells	0.00 (−0.01, 0.01)	0.909	0.912
Activated dendritic cells	0.00 (0.00, 0.00)	0.800	0.912
Resting mast cells	0.01 (−0.07, 0.09)	0.781	0.912
Activated mast cells	0.00 (−0.02, 0.01)	0.574	0.912
Eosinophils	0.01 (−0.02, 0.04)	0.606	0.912

Supplementary Figure S7: Forest plot of the immune cell type fractions of the CIBERSORTx software from the multivariable model corrected for cT and grade compared between dnMBC and eBC. *95% confidence interval was added to show the performance of this model. The coefficient gives the direction of the outcome on the variable. A positive coefficient value indicates a higher outcome in the dnMBC group and vice versa for a negative coefficient value. P-values were calculated with the Wald test and adjusted for multiple testing using the Benjamini-Hochberg method when required. CI: confidence interval; coef: coefficient; dnMBC: de novo metastasized breast tumor group; eBC: non-primary metastatic breast tumor group; FDR: false discovery rate.*
