# Supplementary material for: Differences in the Tumor Molecular and Microenvironmental Landscape between Early (Non-Metastatic) and De Novo Metastatic Primary Luminal Breast Tumors
Source: Cancers (Basel). 2023 Aug 30;15(17):4341. doi: 10.3390/cancers15174341 (PMC10486668; doi:10.3390/cancers15174341)
Supplement: Supplementary file 1 [file cancers-15-04341-s001.zip › Supplementary Table S1.pdf]

Supplementary Table S1:Tumor characteristics (TILs, immune cells, tumor epithelial cells, normal epithelial cells, fibroblasts, and plasma cells) of the study cohort. *Tumor characteristics were determined by an expert breast pathologist via counting the cells of interest microscopically on a H&E stained core biopsy tissue slide. P-values were calculated using a paired Wilcoxon analysis. TILs: Tumor Infiltrating Lymphocytes.*

Variables	Statistics	*De novo* metastasized BC group (dnMBC)	Non-primary metastasized BC group (eBC)	p-value
**TILs (%)**				0.760
	N	32	32	
	Median	2	2	
	Average	7.47	2.90	
	Range	[0.0; 80.0]	[0.0; 9.0]	
Unknown	n/N (%)	3/32 (9%)	1/32 (3%)	
**Immune cells (%)**				0.480
	Median	10	10	
	Average	15	12.10	
	Range	[0.0; 60.0]	[0.0; 30.0]	
Unknown	n/N (%)	3/32 (9%)	1/32 (3%)	
**Tumor epithelial cells (%)**				0.300
	Median	50	50	
	Average	51.03	45.67	
	Range	[30.0; 80.0]	[25.0; 60.0]	
Unknown	n/N (%)	3/32 (9%)	1/32 (3%)	
**Normal epithelial cells (%)**				0.220
	Median	2.50	0	
	Average	5.18	3.39	
	Range	[0.0; 30.0]	[0.0; 15.0]	
Unknown	n/N (%)	4/32 (13%)	1/32 (3%)	
**Fibroblasts (%)**				0.100
	Median	30	35	
	Average	28.96	37.42	
	Range	[0.0; 70.0]	[0.0; 70.0]	
Unknown	n/N (%)	3/32 (9%)	1/32 (3%)	
**Plasma cells (score)** [a]				0.130
0	n/N (%)	23/32 (72%)	25/32 (78%)	
1	n/N (%)	3/32 (9%)	7/32 (22%)	
2	n/N (%)	3/32 (9%)	0/32 (0%)	
3	n/N (%)	1/32 (3%)	0/32 (0%)	
Unknown	n/N (%)	2/32 (6%)	0/32 (0%)	

[a] Categories of the plasma cells were enlisted as 0: none; 1: few scattered; 2: small clusters of 5 plasma cell and at least one; 3: if clusters they are merging (usually plasma cells everywhere)
