# Supplementary material for: Differences in the Tumor Molecular and Microenvironmental Landscape between Early (Non-Metastatic) and De Novo Metastatic Primary Luminal Breast Tumors
Source: Cancers (Basel). 2023 Aug 30;15(17):4341. doi: 10.3390/cancers15174341 (PMC10486668; doi:10.3390/cancers15174341)
Supplement: Supplementary file 1 [file cancers-15-04341-s001.zip › Supplementary Table S10.pdf]

Supplementary Table S10: DEG differences found in the regulatory gene category between dnMBC and eBC tumors. *The gene name, ENSG number, log fold change (logFC), log counts per million (logCPM), log fold change ratio (LR), raw p-value, and FDR-corrected p-value are reported. The base of the log is 2. The direction of logFC is dnMBC vs. eBC. A negative value means a downregulation of the DEG in dnMBC tumors and a positive value means an upregulation of the DEG in dnMBC tumors compared to eBC tumors. The p-values were calculated using paired t-test by using EdgeR. ENSG: Ensembl gene ID; FDR: false discovery rate; snoRNA: small nucleolar RNA.*

						P-values	
**Gene category**	**Gene name**	**ENSG number**	**logFC**	**logCPM**	**LR**	**Raw**	**FDR**
snoRNAs	SNORA38B	ENSG00000200394	-3.632	0.160	64.665	<0.001	<0.001
	SNORA46	ENSG00000207493	-3.398	5.657	78.573	<0.001	<0.001
	SNORA54	ENSG00000207008	-3.125	8.499	65.593	<0.001	<0.001
	SNORA71D	ENSG00000200354	-2.934	7.394	62.719	<0.001	<0.001
	SNORA80B	ENSG00000206633	-4.457	2.313	94.635	<0.001	<0.001
	SNORD10	ENSG00000238917	-3.224	5.153	124.527	<0.001	<0.001
	SNORD114-18	ENSG00000202142	-2.670	-1.200	13.032	<0.001	0.002
	SNORD115-31	ENSG00000202188	-2.994	-2.678	8.483	0.004	0.013
	SNORD116-6	ENSG00000207442	-2.859	-0.454	9.145	0.002	0.010
	SNORD116-8	ENSG00000207093	-2.658	0.927	14.518	<0.001	0.001
	SNORD66	ENSG00000212158	-2.654	2.963	57.725	<0.001	<0.001
	SNORD83A	ENSG00000209482	-3.602	3.011	96.944	<0.001	<0.001
	SNORD94	ENSG00000208772	-3.109	6.450	91.111	<0.001	<0.001
	SNORD3A	ENSG00000202364	-3.616	11.339	62.220	<0.001	<0.001
microRNAs	MIR10A	ENSG00000207777	-3.621	-0.678	24.516	<0.001	<0.001
	MIR10B	ENSG00000207744	-5.263	-0.978	29.651	<0.001	<0.001
	MIR197	ENSG00000207709	-2.542	-0.669	23.147	<0.001	<0.001
	MIR199A2	ENSG00000208024	-3.611	-1.312	29.296	<0.001	<0.001
	MIR200A	ENSG00000207607	-3.166	-0.515	32.431	<0.001	<0.001
	MIR200B	ENSG00000207730	-2.666	-1.228	12.290	<0.001	0.002
	MIR27B	ENSG00000207864	-3.210	-0.167	32.210	<0.001	<0.001
	MIR29b	ENSG00000207790	-2.531	0.060	26.030	<0.001	<0.001
	MIR29c	ENSG00000207966	-4.099	0.206	71.285	<0.001	<0.001
	MIR-34A	ENSG00000207865	-2.568	-0.923	6.702	0.010	0.030
	MIR365A	ENSG00000199130	-4.730	0.438	54.556	<0.001	<0.001
	MIR425	ENSG00000199032	-5.358	-0.840	27.469	<0.001	<0.001
	MIR539	ENSG00000202560	-4.027	-1.463	8.502	0.004	0.013
	MIR544A	ENSG00000207587	-3.197	0.113	14.490	<0.001	<0.001
	MIR545	ENSG00000207820	-3.029	-0.299	22.668	<0.001	<0.001

	MIR98	ENSG00000207787	-3.202	-0.486	40.769	<0.001	<0.001
	MIRLET7A1	ENSG00000199165	-4.079	0.968	71.702	<0.001	<0.001
	MIRLET7D	ENSG00000199133	-4.595	1.434	76.848	<0.001	<0.001
	MIRLET7DHG	ENSG00000230262	-3.736	-0.451	27.177	<0.001	<0.001
	MIRLET7F1	ENSG00000199072	-2.527	0.032	24.516	<0.001	<0.001
	MIRLET7I	ENSG00000199179	-3.911	-0.743	33.773	<0.001	<0.001
Pseudogenes	AC073069.1	ENSG00000237260	-2.618	-0.264	18.149	<0.001	<0.001
	AC084198.1	ENSG00000243396	-2.617	-0.780	16.900	<0.001	<0.001
	AC131392.2	ENSG00000251158	-3.659	0.006	26.196	<0.001	<0.001
	AL732414.1	ENSG00000227934	-2.854	0.442	24.010	<0.001	<0.001
	ALG1L15P	ENSG00000242017	-3.196	-0.695	12.656	<0.001	0.002
	CDH12P2	ENSG00000249230	-2.821	-0.894	15.273	<0.001	0.001
	DUSP5P1	ENSG00000183929	-2.696	-1.592	8.380	0.004	0.014
	DUX4L26	ENSG00000236138	-3.232	1.412	26.191	<0.001	<0.001
	FAM90A20P	ENSG00000233295	-2.513	3.128	30.785	<0.001	<0.001
	FAM90A24P	ENSG00000215354	-4.191	-1.288	6.646	0.010	0.031
	LSP1P2	ENSG00000240665	-2.517	-0.521	10.714	0.001	0.005
	NSUN5P1	ENSG00000223705	-2.640	0.844	37.793	<0.001	<0.002
	NUTM2E	ENSG00000228570	-2.679	-0.576	7.117	0.008	0.025
	OGFOD1P1	ENSG00000231665	-2.603	-0.701	21.116	<0.001	<0.002
	PMS2P6	ENSG00000174384	-2.573	3.045	14.223	<0.001	0.001
	POM121L7P	ENSG00000239511	-2.918	1.142	30.511	<0.001	<0.002
	PSG10P	ENSG00000248257	-3.611	0.418	9.551	0.002	0.008
	RNU1-11P	ENSG00000206702	-3.154	2.690	66.382	<0.001	<0.001
	RNU1-67P	ENSG00000207175	-3.986	0.453	37.232	<0.001	<0.001
	RPS2P32	ENSG00000232818	-2.814	-0.731	21.486	<0.001	<0.001
	RPS7P3	ENSG00000231940	-2.660	0.719	21.315	<0.001	<0.001
	RPSAP39	ENSG00000244002	-2.716	-0.949	15.238	<0.001	0.001
	SERPINH1P1	ENSG00000229207	-2.694	0.020	16.291	<0.001	<0.002
