# Supplementary material for: Differences in the Tumor Molecular and Microenvironmental Landscape between Early (Non-Metastatic) and De Novo Metastatic Primary Luminal Breast Tumors
Source: Cancers (Basel). 2023 Aug 30;15(17):4341. doi: 10.3390/cancers15174341 (PMC10486668; doi:10.3390/cancers15174341)
Supplement: Supplementary file 1 [file cancers-15-04341-s001.zip › Supplementary Table S3.pdf]

Supplementary Table S3: Gene set variation analysis (GSVA) between primary dnMBC tumors vs. eBC. *The hallmark pathways, mean difference, raw p-value, and FDR-corrected p-value are reported. The direction of the mean difference is dnMBC vs. eBC. A negative value means a downregulation of the hallmark pathways in dnMBC tumors and a positive value means an upregulation of the hallmark pathways in dnMBC tumors compared to eBC tumors. The p-values were calculated using a paired-test. FDR: false discovery rate.*

**Hallmarks**	**Mean difference**	**P-values**	
		**Raw**	**FDR**
HALLMARK_HEME_METABOLISM	-0.010	0.018	0.396
HALLMARK_UV_RESPONSE_UP	-0.018	0.020	0.396
HALLMARK_ANDROGEN_RESPONSE	-0.015	0.031	0.396
HALLMARK_PANCREAS_BETA_CELLS	-0.031	0.036	0.396
HALLMARK_KRAS_SIGNALING_DN	-0.012	0.047	0.396
HALLMARK_PROTEIN_SECRETION	-0.013	0.048	0.396
HALLMARK_PI3K_AKT_MTOR_SIGNALING	-0.010	0.056	0.396
HALLMARK_MTORC1_SIGNALING	-0.016	0.087	0.422
HALLMARK_GLYCOLYSIS	-0.010	0.095	0.422
HALLMARK_REACTIVE_OXIGEN_SPECIES_PATHWAY	-0.016	0.097	0.422
HALLMARK_MYC_TARGETS_V1	-0.019	0.098	0.422
HALLMARK_ADIPOGENESIS	-0.011	0.113	0.422
HALLMARK_INTERFERON_ALPHA_RESPONSE	-0.035	0.115	0.422
HALLMARK_PEROXISOME	-0.009	0.118	0.422
HALLMARK_CHOLESTEROL_HOMEOSTASIS	-0.012	0.131	0.435
HALLMARK_FATTY_ACID_METABOLISM	-0.009	0.190	0.591
HALLMARK_ESTROGEN_RESPONSE_LATE	-0.013	0.201	0.591
HALLMARK_P53_PATHWAY	-0.005	0.221	0.613
HALLMARK_ANGIOGENESIS	-0.013	0.255	0.613
HALLMARK_HEDGEHOG_SIGNALING	-0.011	0.266	0.613
HALLMARK_OXIDATIVE_PHOSPHORYLATION	-0.011	0.270	0.613
HALLMARK_MYC_TARGETS_V2	-0.013	0.281	0.613
HALLMARK_INTERFERON_GAMMA_RESPONSE	-0.020	0.282	0.613
HALLMARK_SPERMATOGENESIS	-0.009	0.310	0.629
HALLMARK_IL2_STAT5_SIGNALING	-0.010	0.314	0.629
HALLMARK_MYOGENESIS	-0.010	0.335	0.644
HALLMARK_COAGULATION	-0.009	0.388	0.701
HALLMARK_HYPOXIA	-0.007	0.400	0.701
HALLMARK_XENOBIOTIC_METABOLISM	-0.005	0.406	0.701
HALLMARK_UNFOLDED_PROTEIN_RESPONSE	-0.005	0.442	0.707
HALLMARK_APICAL_JUNCTION	-0.007	0.449	0.707
HALLMARK_MITOTIC_SPINDLE	-0.005	0.452	0.707
HALLMARK_DNA_REPAIR	-0.005	0.466	0.707
HALLMARK_KRAS_SIGNALING_UP	-0.010	0.523	0.767
HALLMARK_TGF_BETA_SIGNALING	0.005	0.537	0.767
HALLMARK_ESTROGEN_RESPONSE_EARLY	-0.007	0.578	0.803
HALLMARK_E2F_TARGETS	-0.010	0.607	0.818
HALLMARK_COMPLEMENT	-0.006	0.629	0.818
HALLMARK_G2M_CHECKPOINT	-0.008	0.638	0.818
HALLMARK_TNFA_SIGNALING_VIA_NFKB	0.005	0.713	0.891

HALLMARK_NOTCH_SIGNALING	0.003	0.779	0.930
HALLMARK_IL6_JAK_STAT3_SIGNALING	0.004	0.789	0.930
HALLMARK_EPITHELIAL_MESENCHYMAL_TRANSITION	0.005	0.816	0.930
HALLMARK_UV_RESPONSE_DN	0.002	0.820	0.930
HALLMARK_BILE_ACID_METABOLISM	-0.001	0.853	0.930
HALLMARK_INFLAMMATORY_RESPONSE	-0.002	0.875	0.930
HALLMARK_APOPTOSIS	-0.001	0.880	0.930
HALLMARK_WNT_BETA_CATENIN_SIGNALING	-0.001	0.907	0.930
HALLMARK_APICAL_SURFACE	-0.001	0.911	0.930
HALLMARK_ALLOGRAFT_REJECTION	0.001	0.973	0.973
