# Supplementary material for: Differences in the Tumor Molecular and Microenvironmental Landscape between Early (Non-Metastatic) and De Novo Metastatic Primary Luminal Breast Tumors
Source: Cancers (Basel). 2023 Aug 30;15(17):4341. doi: 10.3390/cancers15174341 (PMC10486668; doi:10.3390/cancers15174341)
Supplement: Supplementary file 1 [file cancers-15-04341-s001.zip › Supplementary Table S4.pdf]

Supplementary Table S4: Hypoxia-related DEG differences found between dnMBC and eBC tumors. *The gene name, ENSG number, log fold change (logFC), log counts per million (logCPM), log fold change ratio (LR), raw p-value, and FDR-corrected p-value are reported. The base of the log is 2. The direction of logFC is dnMBC vs. eBC. A negative value means a downregulation of the DEG in dnMBC tumors and a positive value means an upregulation of the DEG in dnMBC tumors compared to eBC tumors. The p-values were calculated using paired t-test by using EdgeR. ENSG: Ensembl gene ID; FDR: false discovery rate.*

					P-value	
**Gene name**	**ENSG number**	**logFC**	**logCPM**	**LR**	**Raw**	**FDR**
HIF1A	ENSG00000100644	1.064	7.307	39.985	<0.001	<0.001
MMP2	ENSG00000087245	1.055	8.390	15.816	<0.001	<0.001
P4HA1	ENSG00000122884	0.382	5.941	8.492	0.004	0.013
PLOD2	ENSG00000152952	0.426	6.440	7.622	0.006	0.020
LOX	ENSG00000113083	0.928	3.511	10.528	0.001	0.005
VEGFC	ENSG00000150630	0.677	2.758	7.501	0.006	0.021
ZEB1	ENSG00000148516	0.462	7.148	7.618	0.006	0.020
