# Supplementary material for: Differences in the Tumor Molecular and Microenvironmental Landscape between Early (Non-Metastatic) and De Novo Metastatic Primary Luminal Breast Tumors
Source: Cancers (Basel). 2023 Aug 30;15(17):4341. doi: 10.3390/cancers15174341 (PMC10486668; doi:10.3390/cancers15174341)
Supplement: Supplementary file 1 [file cancers-15-04341-s001.zip › Supplementary Table S6.pdf]

Supplementary Table S6: Baseline neutrophil-to-lymphocyte ratio (NLR), neutrophil counts (percentage and absolute values), and lymphocyte counts (percentage and absolute values) of both study cohorts.

Variables	Statistics	*De novo* metastasized BC group (dnMBC)	Non-primary metastasized BC group (eBC)
**NLR[a]**			
	N	31	32
	Median	3.54	2.55
	Average	3.46	3.17
	Range	[0.58; 6.85]	[1.44; 12.00]
Unknown	n/N (%)	1/32 (3%)	0/32 (0%)
**Neutrophil count (10^9/L)**			
	Median	4.90	4.45
	Average	5.33	4.98
	Range	[1.40; 9.30]	[2.40; 11.50]
Unknown	n/N (%)	1/32 (3%)	0/32 (0%)
**Neutrophil count (%)**			
	Median	70.70	65.95
	Average	67.66	66.08
	Range	[31.00; 82.00]	[49.80; 86.80]
Unknown	n/N (%)	1/32 (3%)	0/32 (0%)
**Lymphocyte count (10^9/L)**			
	Median	1.60	1.70
	Average	1.72	1.78
	Range	[0.90; 3.10]	[0.60; 4.20]
Unknown	n/N (%)	1/32 (3%)	0/32 (0%)
**Lymphocyte count (%)**			
	Median	20.80	25.85
	Average	23.34	24.95
	Range	[11.90; 51.70]	[7.60; 34.00]
Unknown	n/N (%)	1/32 (3%)	0/32 (0%)

[a] NLR was calculated by dividing absolute value of neutrophils (10^9/L) over an absolute value of lymphocytes (10^9/L)
