# Supplementary material for: Differences in the Tumor Molecular and Microenvironmental Landscape between Early (Non-Metastatic) and De Novo Metastatic Primary Luminal Breast Tumors
Source: Cancers (Basel). 2023 Aug 30;15(17):4341. doi: 10.3390/cancers15174341 (PMC10486668; doi:10.3390/cancers15174341)
Supplement: Supplementary file 1 [file cancers-15-04341-s001.zip › Supplementary Table S7.pdf]

Supplementary Table S7: Immunity-related DEG differences found between dnMBC and eBC tumors. *The gene name, ENSG number, log fold change (logFC), log counts per million (logCPM), log fold change ratio (LR), raw p-value, and FDR-corrected p-value are reported. The base of the log is 2. The direction of logFC is dnMBC vs. eBC. A negative value means a downregulation of the DEG in dnMBC tumors and a positive value means an upregulation of the DEG in dnMBC tumors compared to eBC tumors. The p-values were calculated using paired t-test by using EdgeR. ENSG: Ensembl gene ID; FDR: false discovery rate.*

					**P-value**	
**Gene name**	**ENSG number**	**logFC**	**logCPM**	**LR**	**Raw**	**FDR**
CCL11	ENSG00000172156	1.713	0.248	7.326	0.007	0.023
CCL19	ENSG00000172724	1.184	0.579	7.265	0.007	0.023
CCL27	ENSG00000213927	-1.827	-1.190	6.815	0.009	0.028
CCR10	ENSG00000184451	-2.856	-1.405	11.616	0.001	0.003
CCR5	ENSG00000160791	-1.085	1.750	7.775	0.005	0.018
CCR6	ENSG00000112486	-0.981	0.703	6.671	0.010	0.030
CD19	ENSG00000177455	-1.253	1.238	7.898	0.005	0.017
CD27	ENSG00000139193	-0.578	2.770	7.123	0.008	0.025
CD40	ENSG00000101017	-0.631	4.083	11.788	0.001	0.003
CD80	ENSG00000121594	-0.836	1.933	7.408	0.006	0.022
CXCL10	ENSG00000169245	0.683	3.414	5.623	0.018	0.049
CXCL11	ENSG00000169248	1.486	1.152	9.967	0.002	0.007
CXCL13	ENSG00000156234	2.551	3.422	11.721	0.001	0.003
CXCL13	ENSG00000156234	2.551	3.422	11.721	0.001	0.003
CXCL14	ENSG00000145824	0.807	4.339	12.504	<0.001	0.002
CXCL2	ENSG00000081041	2.292	-1.373	8.149	0.004	0.016
CXCL8	ENSG00000169429	1.527	1.115	11.745	0.001	0.003
CXCL9	ENSG00000138755	1.817	2.827	26.340	<0.001	<0.001
IFNAR1	ENSG00000142166	0.816	5.447	43.703	<0.001	<0.001
IFNGR1	ENSG00000027697	0.613	5.956	19.368	<0.001	<0.001
IL13RA1	ENSG00000131724	1.056	6.330	59.113	<0.001	<0.001
IL1R1	ENSG00000115594	0.883	5.802	16.986	<0.001	<0.001
IL1R2	ENSG00000115590	1.935	-0.351	7.070	0.008	0.025
IL6	ENSG00000136244	2.680	-0.358	12.071	0.001	0.003
IL6ST	ENSG00000134352	1.221	9.243	65.429	<0.001	<0.001
ILF2	ENSG00000143621	1.174	6.690	58.601	<0.001	<0.001
JAK1	ENSG00000162434	0.837	7.309	30.882	<0.001	<0.001
STAT1	ENSG00000115415	0.400	9.106	6.780	0.009	0.029
STAT3	ENSG00000168610	0.554	8.765	13.667	<0.001	0.001
